# Muscle transcriptome analysis provides new insights into the growth gap between fast- and slow-growing *Sinocyclocheilus grahami*

**DOI:** 10.3389/fgene.2023.1217952

**Published:** 2023-07-19

**Authors:** Yanhui Yin, Yuanwei Zhang, Zexiang Hua, Anli Wu, Xiaofu Pan, Junxing Yang, Xiaoai Wang

**Affiliations:** ^1^ State Key Laboratory of Genetic Resources and Evolution, Kunming Institute of Zoology, Innovative Academy of Seed Design, Chinese Academy of Sciences, Kunming, Yunnan, China; ^2^ Yunnan Key Laboratory of Plateau Fish Breeding, Kunming Institute of Zoology, Chinese Academy of Sciences, Kunming, Yunnan, China; ^3^ Yunnan Engineering Research Center for Plateau-Lake Health and Restoration, Kunming Institute of Zoology, Chinese Academy of Sciences, Kunming, Yunnan, China; ^4^ Kunming College of Life Science, University of Chinese Academy of Sciences, Beijing, China; ^5^ Fishery Technology Extension Station of Yunnan, Kunming, Yunnan, China

**Keywords:** growth, metabolism, collagen synthesis, WGCNA, crucial genes

## Abstract

*Sinocyclocheilus grahami* is an economically valuable and famous fish in Yunnan Province, China. However, given its slow growth (40 g/2 years) and large growth differences among individuals, its growth performance needs to be improved for sustainable future use, in which molecular breeding technology can play an important role. In the current study, we conducted muscle transcriptomic analysis to investigate the growth gaps among individuals and the mechanism underlying growth within 14 fast- and 14 slow-growth *S. grahami*. In total, 1,647 differentially expressed genes (DEGs) were obtained, including 947 up-regulated and 700 down-regulated DEGs in fast-growth group. Most DEGs were significantly enriched in ECM-receptor interaction, starch and sucrose metabolism, glycolysis/gluconeogenesis, pyruvate metabolism, amino acids biosynthesis and metabolism, peroxisome, and PPAR signaling pathway. Some genes related to glycogen degradation, glucose transport, and glycolysis (e.g., *adipoq*, *prkag1*, *slc2a1*, *agl*, *pygm*, *pgm1*, *pfkm*, *gapdh*, *aldoa*, *pgk1*, *pgam2*, *bpgm*, and *eno3*) were up-regulated, while some genes related to fatty acid degradation and transport (e.g., *acox1*, *acaa1*, *fabp1b.1*, *slc27a1*, and *slc27a2*) and amino acid metabolism (e.g., *agxt*, *shmt1*, *glula*, and *cth*) were down-regulated in the fast-growth group. Weighted gene co-expression network analysis identified *col1a1*, *col1a2*, *col5a1*, *col6a2*, *col10a1*, *col26a1*, *bglap*, and *krt15* as crucial genes for *S. grahami* growth. Several genes related to bone and muscle growth (e.g., *bmp2*, *bmp3*, *tgfb1*, *tgfb2*, *gdf10,* and *myog*) were also up-regulated in the fast-growth group. These results suggest that fast-growth fish may uptake adequate energy (e.g., glucose, fatty acid, and amino acids) from fodder, with excess energy substances used to synthesize collagen to accelerate bone and muscle growth after normal life activities are maintained. Moreover, energy uptake may be the root cause, while collagen synthesis may be the direct reason for the growth gap between fast- and slow-growth fish. Hence, improving food intake and collagen synthesis may be crucial for accelerating *S. grahami* growth, and further research is required to fully understand and confirm these associations.

## 1 Introduction

*Sinocyclocheilus grahami* (Cypriniformes, Cyprinidae) is an endemic fish species in China, with restricted distribution in Dianchi Lake and surrounding streams on the Yunnan Plateau (Zhao and Zhang, 2009). As one of the “Four Famous Fishes” in Yunnan, *S. grahami* is an economically valuable species known for its excellent quality and higher crude protein (∼20%), essential amino acids (∼18%) and polyunsaturated fatty acids (∼0.34%) content than *Ctenopharyngodon idellus*, *Hypophthalmichthys nobilis*, or *Cyprinus carpio* (Zhao et al., 2013), and possesses huge breeding potential, especially in freshwater aquaculture. From the 1960s, the species became highly endangered due to habitat destruction, water pollution, and alien species invasion (Yang et al., 2007). As such, over the past 2 decades, our team has successfully established an artificial breeding program to ensure the survival of the species and lay a foundation for its production, resulting in the creation of a new national breed (“*S. grahami*, Bayou No. 1”, hereafter *S. grahami*) with accelerated growth and weakened intermuscular bones via four generations of artificial selection (Yang et al., 2007; Pan et al., 2009; Yin et al., 2021). However, slow growth (40 g/2 years, 2 years = mature age) and growth gaps among individuals remain problematic, and further selective breeding is required to obtain a faster and more stable growing strain.

With the rapid development of molecular biology and sequencing technology, modern breeding techniques attempt to target the regulatory genes underpinning desired phenotypes and achieve superior varieties via the selection or manipulation of these genes, i.e., molecular-assisted breeding (Eze, 2019; Liu et al., 2022). Therefore, it has become increasingly important to understand the regulatory mechanisms and major genes behind desired phenotypes.

Although growth is a vital characteristic of farmed species, it is a complex trait influenced by many genes with minor effects. As such, the genetic mechanisms underlying growth remain unclear, although various relevant genes have been identified, including growth axis-related genes [growth hormone (*gh*), growth hormone receptor (*ghr*), insulin-like growth factor I (*igf1*), insulin-like growth factor II (*igf2*), somatostatin (*sst*)] and muscle growth regulating genes [myostatin (*mstn*), myogenic regulatory factors (*mrfs*)] and appetite, food intake regulate genes [melanocortin receptor-4 (*mc4r*), ghrelin (*ghrl*)] (De-Santis and Jerry, 2007; Blanco et al., 2017; Baldini and Phelan, 2019). Nevertheless, many genes related to growth remain unresolved, and the mechanisms for growth differ in different species (Laghari et al., 2014; Yu et al., 2016; Chen et al., 2022). Hence, mechanistic analysis of *S. grahami* is required to better guide breeding.

Fish growth can be achieved via skeletal muscle growth, primarily determined by hyperplasia and hypertrophy of muscle fibers (muscle cells) (Stoiber et al., 2002; Johnston, 2006; Fuentes et al., 2013). Muscle constitutes 50%–70% of body weight of most commercially important fish species and is the main consumed product (Li et al., 2019). Therefore, muscle growth plays a critical role in fish growth. Transcriptomic analysis plays a significant role in all fields of biological research and is widely used to study gene expression profiles and functional mechanisms of genotypes (Klopfleisch and Gruber, 2012; Papatheodorou et al., 2015). For instance, muscle transcriptomic studies have identified several genes correlated with growth in a variety of fish species, including *Micropterus salmoides* (Li et al., 2017), *Ctenopharyngodon idella* (Lu et al., 2020), *Schizothorax prenanti* (Li et al., 2019), and *Mylopharyngodon piceus* (Zhang et al., 2020).

Here, we focused on exploring the molecular mechanisms and major genes underlying growth of *S. grahami*, and aimed to verify differences in gene expression profiles between fast- and slow-growth fish and identify key genes involved in body length/weight based on muscle transcriptomic analysis. We hope that our study can provide valuable information for further studying of growth mechanisms and breeding strategies in *S. grahami* and other farmed species.

## 2 Materials and methods

### 2.1 Ethics statement

All research protocols and treatments of experimental fish were reviewed and approved by the Internal Review Board of the Kunming Institute of Zoology (KIZ), Chinese Academy of Sciences (CAS), China (approval ID: IACUC-PA-2021-07-053).

### 2.2 Preliminary study

To determine the effect of random variables caused by rearing environment and select the most appropriate samples for next analysis, a preliminary study was conducted. We carried out a bulk RNA-seq strategy for extremely large and small size samples in a sibling population (generated from one female × one male) and a random population (generated from multiple females × males) from the farmed “*S. grahami*, Bayou No. 1”. After RNA-seq data analysis, we observed that the major differentially expressed genes (DEGs) were similar between the two populations (Supplementary Figure S2). It is evident that when the rearing conditions are consistent, the random variables caused by rearing environment can be negligible. Therefore, to further investigate the mechanisms underlying growth, in the current study, we selected the random population (diverse genetic backgrounds) cultivated in one tank to perform further analysis. The detailed information for preliminary study was provided in Supplementary Material.

### 2.3 Fish cultivation, sample collection, and sequencing of *S. grahami*

Fish were obtained from the Endangered Fish Conservation Center (EFCC) of the Kunming Institute of Zoology (KIZ), Chinese Academy of Sciences, Kunming, Yunnan, China. In February 2018, a random population (generated from multiple females × males, ∼20,000 individuals) of farmed *S. grahami* was constructed using artificial reproduction. The resulting offspring were cultivated in a 3 m × 4 m × 1.5 m pond, with the water temperature, dissolved oxygen (DO) and PH levels maintained near 22°C ± 1°C, 6.5 mg/L and 8.0, respectively. One-third of water in pond was changed with fresh water daily. They were fed twice a day (fodder volume 3% of fish weight) to apparent satiation by a commercial diet (protein 40%, lipid 18%, Specialized and high-end feed for freshwater fish, Tongwei Group) for 8 months at the EFCC. We then separated individuals into large-, medium-, and small-sized groups (in different buckets) according to body size. To further observe the growth gap and minimize the effect of environmental random variables for our results, we transported 500 individuals (200 extremely large, 200 extremely small, and 100 medium-sized individuals) labeled with visible implant elastomer (VIE) of different colors to one fish tank (1 m × 0.6 m × 1 m) in the laboratory at KIZ. The rearing conditions and feeding regime were consistent with previous setup. After 2 months of regular feeding, we measured body weight and body length separately, and the growth gap between extremely large, medium-sized and extremely small individuals persists throughout the experiment. Therefore, we selected 14 extremely large and 14 extremely small individuals as the two extreme bulks of growth.

Fish samples from the two extreme bulks were first euthanized using MS-222. Muscle tissues of each individual were collected and kept under sterile conditions. Subsequently, total RNA was extracted using an RNA Purification Kit (Omega BioTek, United States) in accordance with the manufacturer’s instructions. For each sample, RNA concentration and quality were measured using a Nanophotometer (Implen, Germany) and Agilent 2100 Bioanalyzer (Agilent Technologies Inc., United States), respectively. High-quality samples (OD260/280 ≥ 1.8, OD260/230 ≥ 1.8) were labelled into a paired-end 150-bp library and sequenced using the Illumina Hiseq X-Ten platform.

### 2.4 Transcript-level gene expression analysis and functional enrichment of differentially expressed genes (DEGs)

Raw RNA sequencing (RNA-seq) reads from each sample were filtered using FastQC (v0.11.8) and Trimmomatic (v0.38). Clean reads were aligned to the reference genome (GenBank: GCA_001515645.1) using Hisat2 (v2.1.0) with default parameters. The unique mapped reads of each sample were used to calculate fragments per kilobase of exon model per million mapped fragments (FPKM) using Cufflinks (v2.2.1) with default settings. According to the gene expression level of each sample, package DEseq2 in R (v4.0.5) was used to detect significant DEGs, with |log2 (fold-change)| ≥ 1 and adjusted *p* ≤ 0.05 applied as filtering thresholds. To further clarify the functions of DEGs, Gene Ontology (GO) and Kyoto Encyclopedia of Genes and Genomes (KEGG) pathway enrichment analyses were performed using DAVID (https://david.ncifcrf.gov/summary.jsp) and the clusterProfiler package in R (v4.0.5).

### 2.5 Weighted gene co-expression network analysis (WGCNA)

The FPKM of all genes was used to build unsigned co-expression networks using the WGCNA package in R (v4.0.5) (Langfelder and Horvath, 2008). We first used the pickSoftThreshold function in the WGCNA package to calculate the weighting coefficient *β* to ensure that the resulting network was close to scale-free topology (linear regression model satisfies R^2^ = 0.85 as a threshold). The Pearson correlation matrix was then used to analyze the co-expression of the paired genes, and network construction was performed using the one-step function (blockwiseModules) in the WGCNA package with parameters “maxBlockSize = nGenes, TOMType = ‘unsigned’, minModuleSize = 30, reassignThreshold = 0, mergeCutHeight = 0.25, corType = ‘pearson’ ”. Next, the correlation coefficient between the module eigenvector (module eigengene, ME) and different influencing factors was calculated to determine the module most highly related to the phenotype. Module membership ≥ 0.8 and gene significance ≥ 0.2 were set as the threshold of hub genes screened in the optimal-related module. Cytoscape (v3.7.2) was used to analyze the degree of genes and construct the visualization network.

### 2.6 Quantitative real-time PCR

To validate the transcriptome data, ten DEGs were randomly chosen and their mRNA levels were assessed using qRT-PCR (quantitative real-time PCR) in six fast-growth and six slow-growth samples (randomly selected). All primers designed by Primer-BLAST (https://www.ncbi.nlm.nih.gov/tools/primer-blast/) according to CDS (Coding Sequence) sequences of *S. grahami* from the National Center for Biotechnology Information (NCBI) database. For qRT-PCR, 0.25 µg of total RNA was used for cDNA synthesis with PrimeScript™ RT Reagent Kit with gDNA Eraser (Takara, Japan) based on manuals. The two-step qRT-PCR program included enzyme activation at 95°C (30 s) and 40 cycles at 95°C (5 s), 60°C (30 s) was performed with TB Green^®^ Premix Ex Taq™ II (TaKaRa, Japan) using the CFX Connect Real-Time System (BioRad, United States). PCR amplification of all samples was performed in triplicate. Eukaryotic translation elongation factor 2 (*Eef2*) was used as the reference gene to calculate the relative expression levels mainly because of its stability, which the CT values are similar in all samples (Zhang et al., 2017; Zhang et al., 2019). Fold changes in gene expression were calculated using the 2^−ΔΔCT^ method (Livak and Schmittgen, 2001).

### 2.7 Statistical analysis

Statistical analysis was performed with Excel 2010 and SPSS 25.0 (SPSS, United States). All data were presented as mean ± standard deviation (SD). Significant differences were analyzed via one-way ANOVA (analysis of variance) and significance was accepted at the level of *p* < 0.05.

## 3 Results

### 3.1 Sample collection and sequencing

**FIGURE 1:**
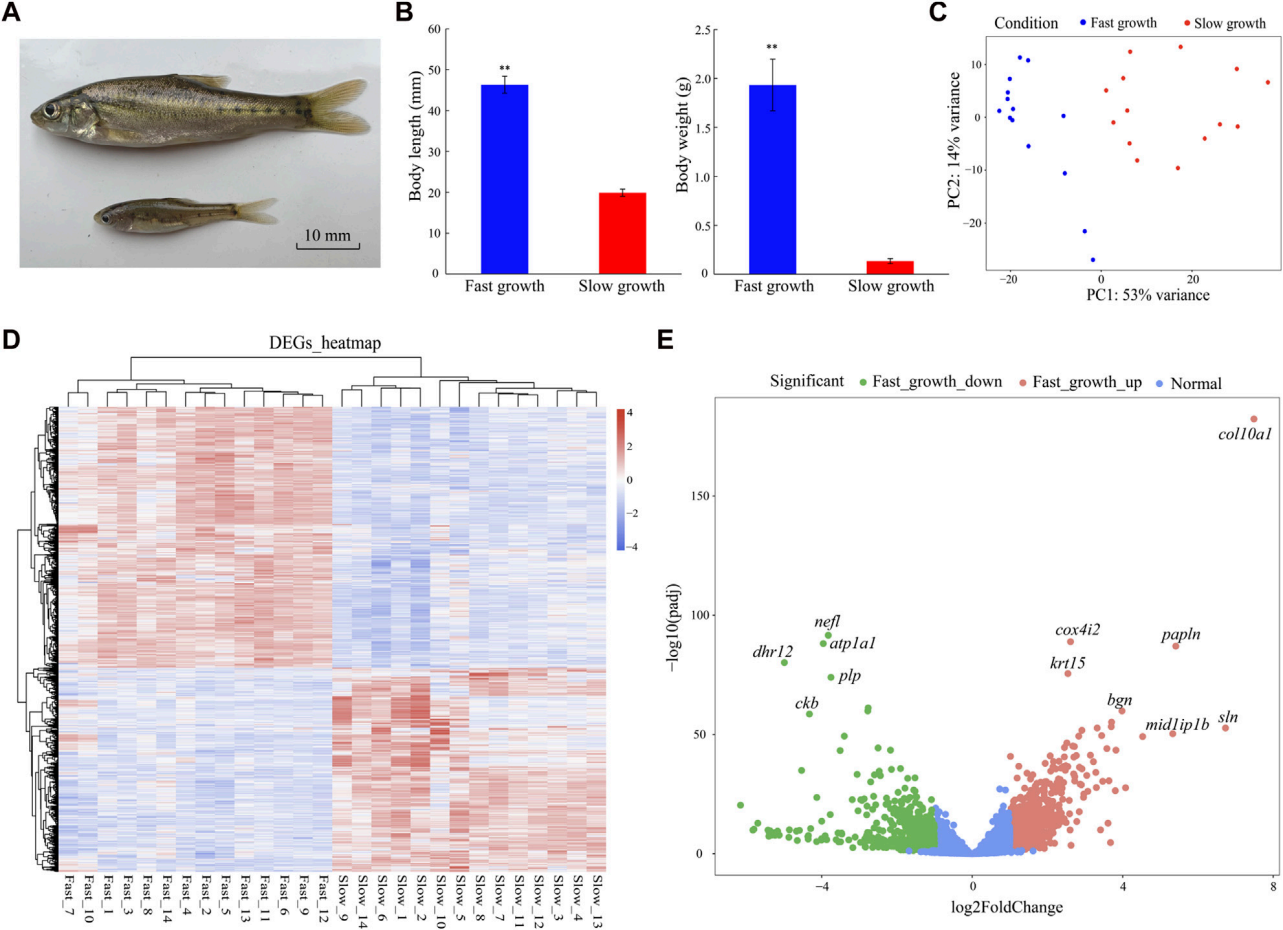
Phenotypes and DEGs of fast- and slow-growth *S. grahami*. **(A)** Living specimen of fast- and slow-growth *S. grahami*. **(B)** Body length/weight variations in fast- and slow-growth groups. *P* < 0.01 are represented with two asterisks. **(C)** PCA of correlation between phenotype and gene expression in fast- and slow-growth groups. **(D)** Heatmap of DEGs for fast- and slow-growth groups. **(E)** Volcano plot for fast- and slow-growth groups.

In total, 28 individuals at the same developmental period (10 months old) but with different growth rates (14 fastest growing individuals, body length: 46.33 ± 2.08 mm, body weight 1.93 ± 0.26 g; 14 slowest growing individuals, body length: 19.88 ± 0.86 mm, body weight: 0.13 ± 0.02 g) were selected for analysis. Body length and weight were significantly different between the fast-growth and slow-growth groups (*p* < 0.01) (Figures 1A, B).

The 28 RNA samples were sequenced using the Illumina Hiseq X-Ten platform. After quality trimming, a total of 781,181,070 clean reads (150 bp) were generated for analysis (Supplementary Table S1). Among them, 390,057,252 reads were from the fast-growth individuals and 391,123,818 were from the slow-growth individuals. The Q30 range was 95.25%–95.78% for all individuals, indicating that data quality of each sample was sufficient for the following analyses. Principal component analysis (PCA) was performed to explore the relationship between gene expression and body length/weight before differential expression analysis. Results showed that the fast- and slow-growth groups could be differentiated by PC1 (explaining 53% of the variance) (Figure 1C), indicating that PC1 was correlated with body length/weight, and the following analyses for major DEGs was feasible.

### 3.2 Differential expression and functional enrichment analysis

In total, 1,647 DEGs (two-fold change in expression and adjusted *p* < 0.05) were identified in the fast- versus slow-growth groups, including 947 up-regulated and 700 down-regulated DEGs in the fast-growth group (Figures 1D, E). Based on the DEGs heatmap, DEGs expression were significantly different between the two groups, but were consistent in the 14 fast-growth samples and 14 slow-growth samples, indicating no significant differences within groups and that the DEGs were suitable for subsequent analyses (Figure 1D). The markedly up-regulated genes for fast-growth group included collagen alpha-1(X) chain (*col10a1*), papilin (*papln*), sarcolipin (*sln*), mid1-interacting protein 1-B (*mid1ip1b*), biglycan (*bgn*), cytochrome c oxidase subunit 4 isoform 2 (*cox4i2*), and keratin, type I cytoskeletal 15 (*krt15*), while the markedly down-regulated genes included neurofilament light polypeptide (*nefl*), dehydrogenase/reductase SDR family member 12 (*dhr12*), sodium/potassium-transporting ATPase subunit alpha-1 (*atp1a1*), myelin proteolipid protein (*plp*), and creatine kinase B-type (*ckb*) (Figure 1E).

**FIGURE 2:**
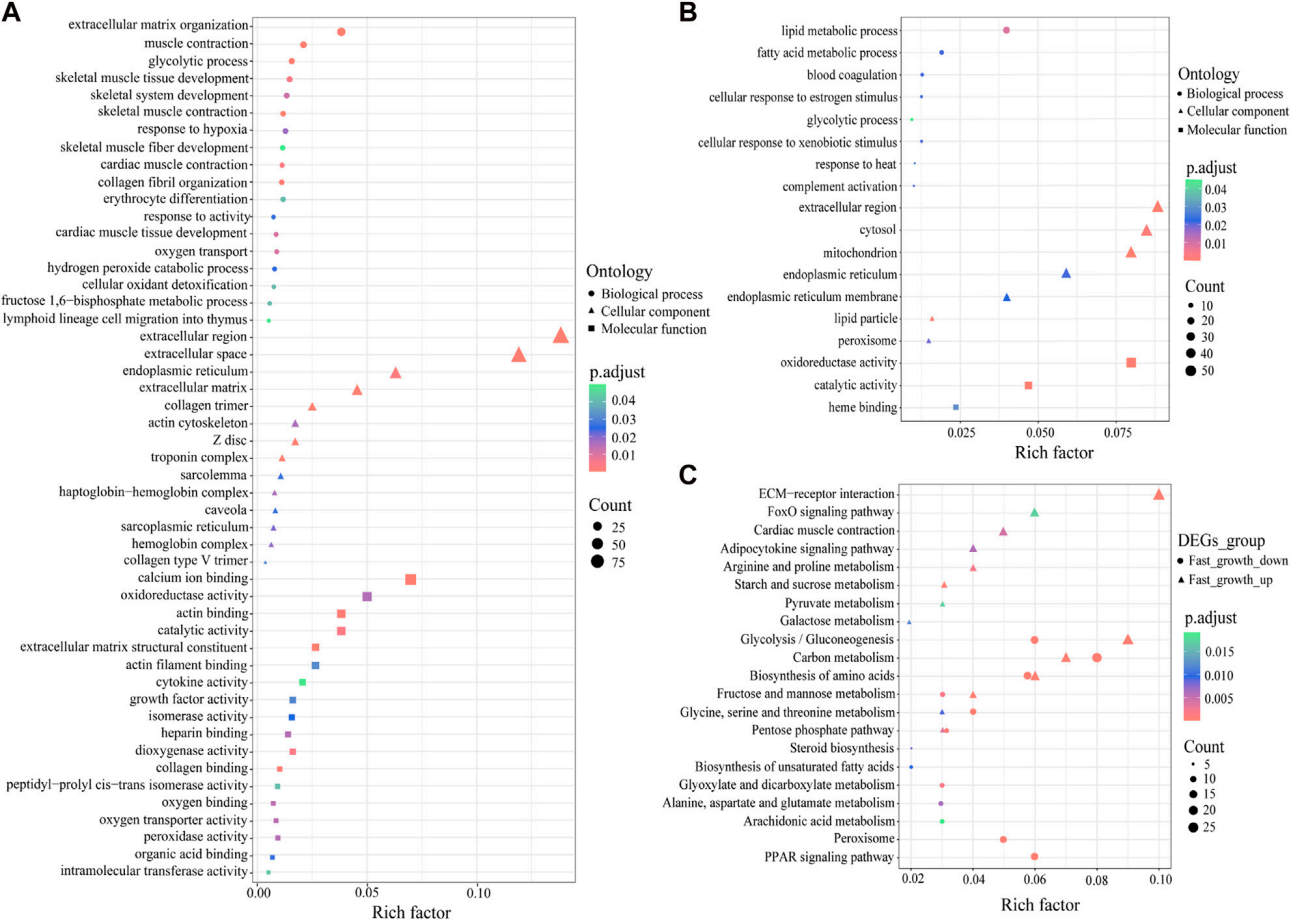
GO and KEGG enrichment analysis of DEGs. **(A)** GO enrichment analysis of up-regulated DEGs. **(B)** GO enrichment analysis of down-regulated DEGs. **(C)** KEGG enrichment analysis of up- and down-regulated DEGs. *X*-axis represents the rich factor, which reflects the degree of enrichment of DEGs in each KEGG pathway.

To understand the functions of the DEGs, we performed GO enrichment analysis. The 947 up-regulated DEGs were classified into 37 GO terms (adjusted *p* < 0.05) (Figure 2A), including extracellular matrix organization (GO:0030198), glycolytic process (GO:0006096), skeletal muscle tissue development (GO:0007519), skeletal system development (GO:0001501), collagen fibril organization (GO:0030199), skeletal muscle fiber development (GO:0048741), and growth factor activity (GO:0008083). In addition, the 700 down-regulated DEGs were classified into nine GO terms (adjusted *p* < 0.05) (Figure 2B), including lipid metabolic process (GO:0006629), fatty acid metabolic process (GO:0006631), glycolytic process (GO: 0006096), oxidoreductase activity (GO:0016491), and catalytic activity (GO:0003824).

To further identify the biological pathways that regulate growth in *S. grahami*, we performed KEGG pathway analysis of the up- and down-regulated DEGs (adjusted *p* < 0.05). Results showed that the up-regulated DEGs were primarily enriched in ECM-receptor interaction, glycolysis/gluconeogenesis, carbon metabolism, biosynthesis of amino acids, FoxO signaling pathway, cardiac muscle contraction, arginine and proline metabolism, adipocytokine signaling pathway, starch and sucrose metabolism, glycine, serine, and threonine metabolism, galactose metabolism, pyruvate metabolism, pentose phosphate pathway, and fructose and mannose metabolism (Figure 2C). In addition, several down-regulated DEGs were also enriched in glycolysis/gluconeogenesis, biosynthesis of amino acids, fructose and mannose metabolism, carbon metabolism, pentose phosphate pathway, glycine, and serine and threonine metabolism (Figure 2C). Other down-regulated DEGs were enriched in the PPAR signaling pathway, peroxisome, glyoxylate and dicarboxylate metabolism, alanine, aspartate, and glutamate metabolism, steroid biosynthesis, biosynthesis of unsaturated fatty acids, and arachidonic acid metabolism (Figure 2C).

### 3.3 Weighted gene co-expression network analysis

**FIGURE 3:**
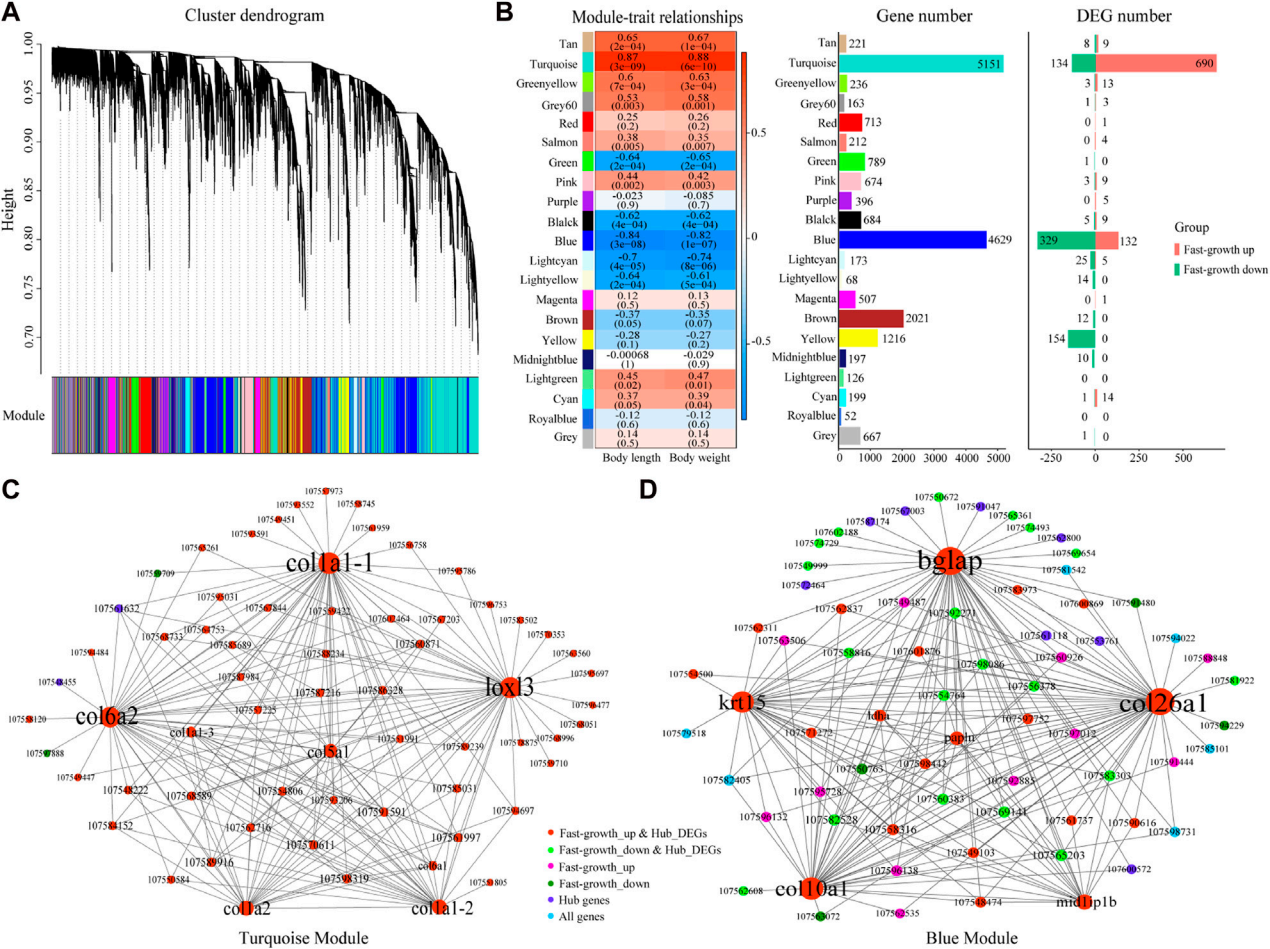
WGCNA for *S. grahami.* **(A)** Average linkage clustering tree (dendrogram) based on topological overlap distance in gene expression profiles in muscle datasets. Branches of dendrogram correspond to modules, shown in “module” color bar below dendrogram. **(B)** Correlation between module eigengenes and phenotype and module genes. Left: Correlation between module eigengenes and phenotype. Each row corresponds to a module identified on the left side by its color. Each column corresponds to a phenotype. Each cell reports Pearson correlation between module eigengene and phenotype. Cells are color-coded using correlation values according to color scale on the right; positive correlations are in red and negative correlations are in blue. Middle: Gene number in each module. Right: DEG number in each module. **(C)** Network view of turquoise module. Node are labeled with gene symbols, colored according to gene type, and sized according to gene degree. **(D)** Network view of blue module. Node are labeled with gene symbols, colored according to gene type, and sized according to gene degree.

To better understand the relationships between genes and phenotypes, we used 19,094 genes for WGCNA and a soft-power threshold of *β* = 6 for further analysis. In total, 20 modules were classified with module sizes ranging from 52 to 5,151, with 667 genes not assigned to any module (Figures 3A, B). The most abundant module was the turquoise module, containing 5,151 genes, including 690 up-regulated and 134 down-regulated DEGs (Figure 3B), followed by the blue module, containing 4,629 genes, including 132 up-regulated and 329 down-regulated DEGs (Figure 3B). Based on the correlation coefficient of modules and sample body weight/length, the turquoise (*R* > 0.87, *p* < 0.05) and blue (*R* < −0.82, *p* < 0.05) modules were significantly positively and negatively correlated with body length/weight, respectively (Figure 3B). Based on module membership ≥ 0.8 and gene significance ≥ 0.2, 739 and 529 genes were identified as hub genes in the turquoise and blue modules, respectively.

As many hub genes were found under the threshold criteria, we identified crucial genes according to the degree of node connection. In the turquoise module, 65 genes with the strongest interaction (top 200 weight pairs, weight value > 0.5403) were chosen for network construction. Lysyl oxidase homolog 3 (*loxl3*), collagen alpha-2(VI) chain (*col6a2*), collagen alpha-1(I) chain (*col1a1*), collagen alpha-1(V) chain (*col5a1*), and collagen alpha 2(I) chain (*col1a2*), which showed the highest degree of node connection (degree ≥ 22), were up-regulated in the fast-growth group, and positively correlated with body weight/length (Figure 3C). Simultaneously, in the blue module, 69 genes with the strongest interaction (top 200 weight pairs, weight value > 0.5225) were chosen for network construction. Osteocalcin (*bglap*), collagen alpha-1 (XXVI) chain (*col26a1*), collagen alpha-1(X) chain (*col10a1*), keratin, type I cytoskeletal 15 (*krt15*), and mid1-interacting protein 1-B (*mid1ip1b*), which showed the highest degree of node connection (degree ≥ 18), were up-regulated in the fast-growth group, and positively correlated with body weight/length (Figure 3D). Thus, these identified genes may be crucial genes for *S. grahami* growth in the turquoise and blue modules.

### 3.4 Validation by quantitative real-time PCR

**FIGURE 4:**
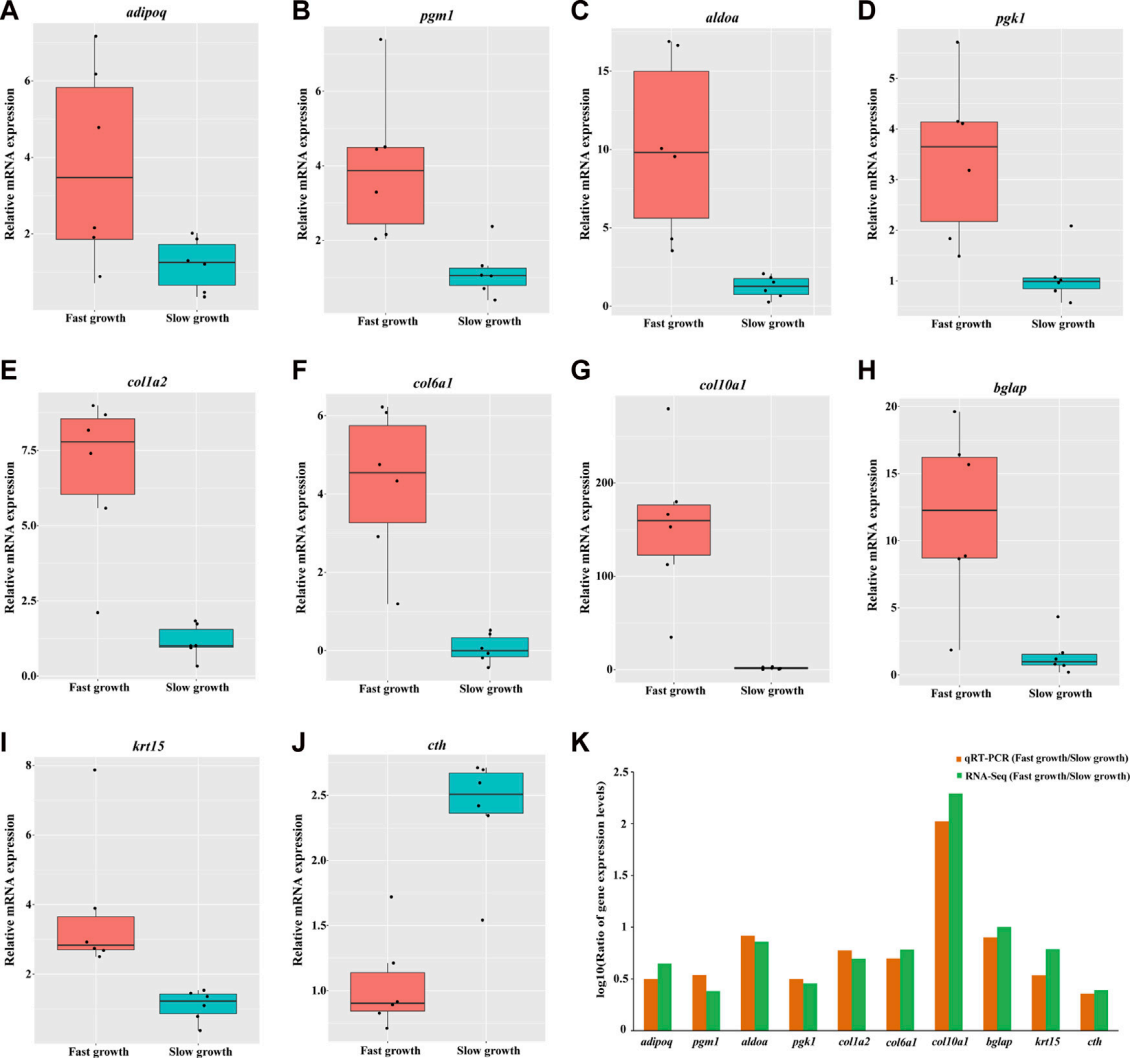
Validation of gene expression for 10 DEGs by quantitative real-time PCR. The relative mRNA expression levels of *adipoq*, *pgm1*, *aldoa*, *pgk1*, *col1a2*, *col6a1*, *col10a1*, *bglap*, *krt15*, *cth* generated by qRT-PCR are depicted in A, B, C, D, E, F, G, H, I, respectively. The red and cyan boxes represent the fast- and slow-growth groups, respectively. Each black dot represents the relative mRNA expression level of a sample. The logarithmic scale (log10) for the ratio of DEGs expression levels between fast- and slow-growth groups for qRT-PCR and RNA-seq were depicted in K.

Ten DEGs were chosen for qRT-PCR to validate the expression pattern observed in transcriptome data. Figure 4 displays the relative expression levels of *adipoq*, *pgm1*, *aldoa*, *pgk1*, *col1a2*, *col6a1*, *col10a1*, *bglap*, *krt15*, *cth* in both fast- and slow-growth groups. The ratio of DEGs expression levels between the fast and slow-growth groups were calculated for qRT-PCR and RNA-seq data, respectively (Figure 4K). The result indicates the expression pattern observed in qRT-PCR is consistent with that observed in the RNA-seq data (Supplementary Figure S3). Moreover, these expression patterns were consistent with that observed in the bulk RNA-Seq data for preliminary study (Supplementary Figure S4), strongly indicate the reliability of the results in our study. The qRT-PCR primers of these DEGs were provided in Supplementary Table S2.

## 4 Discussion

As a critical economic trait, growth is important for the development of aquaculture. A growing number of transcriptomic studies have identified growth mechanism diversity in species, tissues, and environments (Fu et al., 2019; Prieto et al., 2019; Lu et al., 2020; Wang et al., 2021). In our study, 1,647 DEGs (947 up-regulated and 700 down-regulated DEGs in the fast-growth group) were obtained from muscle tissue between the fast- and slow-growth groups. Most DEGs were significantly enriched in metabolic pathways, such as starch and sucrose metabolism, glycolysis/gluconeogenesis, pyruvate metabolism, glycine, serine and threonine metabolism, arginine and proline metabolism, biosynthesis of amino acids, peroxisome, and PPAR signaling pathway (Figure 2C). These findings are similar to those in previous studies on *C. idella* (Lu et al., 2020), *Eriocheir sinensis* (Wang et al., 2021), and *Paramisgurnus dabryanus* (Zhao et al., 2021), suggesting that metabolism plays a crucial role in growth, and the fast-growth group exhibit higher expression of some metabolic genes than slow-growth group.

**FIGURE 5:**
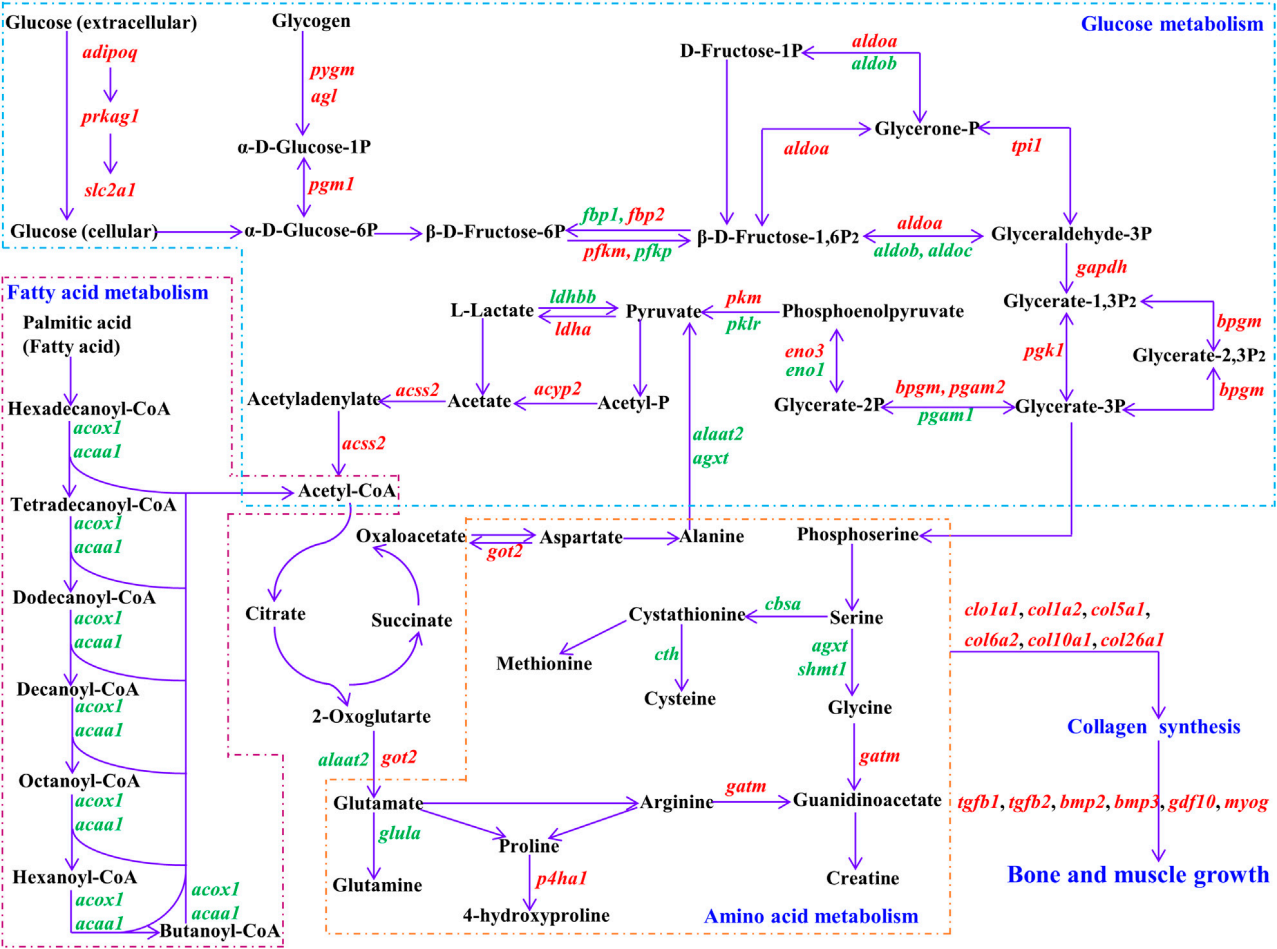
Regulatory networks for vital DEGs. Black represents substrate for biological process. Purple arrow represents direction of reaction. Red represents up-regulated DEGs, green represents down-regulated DEGs in fast-growth group. *adipoq*: adiponectin; *prkag1*: 5′-AMP-activated protein kinase subunit gamma-1; *slc2a1*: solute carrier family 2, facilitated glucose transporter member 1; *pygm*: glycogen phosphorylase, muscle form; *agl*: glycogen debranching enzyme; *pgm1*: phosphoglucomutase-1; *fbp1*: fructose-1,6-bisphosphatase 1; *fbp2*: fructose-1,6-bisphosphatase 2; *pfkm*: ATP-dependent 6-phosphofructokinase, muscle type; *pfkp*: ATP-dependent 6-phosphofructokinase, platelet type; *aldoa*: fructose-bisphosphate aldolase A; *aldob*: aldolase b, fructose-bisphosphate; *aldoc*: fructose-bisphosphate aldolase C; *tpi1*: triosephosphate isomerase 1; *gapdh*: glyceraldehyde-3-phosphate dehydrogenase; *pgk1*: phosphoglycerate kinase 1; *bpgm*: 2,3-bisphosphoglycerate mutase; *pgam1*: phosphoglycerate mutase 1; *pgam2*: phosphoglycerate mutase 2; *eno1*: alpha-enolase; *eno3*: beta-enolase; *pkm*: pyruvate kinase PKM; *pklr*: pyruvate kinase L/R; *ldha*: L-lactate dehydrogenase A chain; *ldhbb*: lactate dehydrogenase Bb; *acyp2*: acylphosphatase 2; *acss2*: acetyl-coenzyme A synthetase**,** cytoplasmic; *acox1*: peroxisomal acyl-coenzyme A oxidase 1; *acaa1*: 3-ketoacyl-CoA thiolase B, peroxisomal; *got2*: aspartate aminotransferase, mitochondrial; *agxt*: serine-pyruvate aminotransferase, mitochondrial; *alaat2*: alanine aminotransferase 2; *glula*: glutamate-ammonia ligase (glutamine synthase); *cbsa*: cystathionine beta-synthase a; *cth*: cystathionine gamma-lyase; *shmt1*: serine hydroxymethyltransferase 1; *gatm*: glycine amidinotransferase, *mitochondrial*; *p4ha1*: prolyl 4-hydroxylase subunit alpha-1; *col1a1*: collagen alpha-1(I) chain; *col1a2*: collagen alpha 2(I) chain; *col5a1*: collagen alpha-1(V) chain; *col6a2*: collagen alpha-2(VI) chain; *col10a1*: collagen alpha-1(X) chain; *col26a1*: collagen alpha-1 (XXVI) chain; *tgfb1*: transforming growth factor beta-1; *tgfb2*: transforming growth factor beta-2; *bmp2*: bone morphogenetic protein 2; *bmp3*: bone morphogenetic protein 3; *gdf10*: growth/differentiation factor 10; *myog*: myogenin.

Achieving somatic growth requires depletion of available nutrients and energy acquired from the environment, which are converted into cellular and tissue components through metabolic cellular reactions (Sousa et al., 2010; Lukas et al., 2011; Canosa and Bertucci, 2020). Glucose and fatty acids are the most important sources of energy for animal growth (Judge and Dodd, 2020). Animals preferentially use dietary glucose and fatty acids for energy supply. When the body enters a long period of fasting or starvation, stored glycogen, fatty acids, and protein will be successively decomposed and utilized to maintain normal life activities (Judge and Dodd, 2020). In our study, adiponectin (*adipoq*), 5′-AMP-activated protein kinase subunit gamma-1 (*prkag1*), and solute carrier family 2, facilitated glucose transporter member 1 (*slc2a1*) were up-regulated and enriched in the adipocytokine signaling pathway. Studies have shown that adiponectin (encoded by *adipoq*) can activate AMP-activated protein kinase (AMPK) subunits (encoded by *prkag1*), thereby directly regulating glucose metabolism and insulin sensitivity (Yamauchi et al., 2002; Schönke et al., 2015). Adiponectin is also an important appetite regulator, modulating energy homeostasis by increasing appetite, boosting substrate storage, and decreasing energy expenditure (Wolf, 2003; Jeon et al., 2021). Glucose transporter 1, a uniporter protein encoded by *slc2a1*, facilitates glucose diffusion across the cell membrane, regulating the first limiting step (glucose transport into cells) for glucose homeostasis (Yan, 2017; Coudert et al., 2018). Moreover, several genes involved in glycogen degradation and glycolysis were up-regulated in the fast-growth group (Figure 5), including glycogen debranching enzyme (*agl*), glycogen phosphorylase, muscle form (*pygm*), phosphoglucomutase-1 (*pgm1*), ATP-dependent 6-phosphofructokinase, muscle type (*pfkm*), fructose-bisphosphate aldolase A (*aldoa*), glyceraldehyde-3-phosphate dehydrogenase (*gapdh*), phosphoglycerate kinase 1 (*pgk1*), phosphoglycerate mutase 2 (*pgam2*), 2,3-bisphosphoglycerate mutase (*bpgm*), beta-enolase (*eno3*), pyruvate kinase PKM (*pkm*), L-lactate dehydrogenase A chain (*ldha*), and acetyl-coenzyme A synthetase, cytoplasmic (*acss2*). High expression of these genes indicates that glucose utilization ability is higher in the fast-growth *S. grahami* fish.

In addition, several genes related to glycolysis were down-regulated in fast-growth *S. grahami* (Figure 5), including ATP-dependent 6-phosphofructokinase, platelet type (*pfkp*), aldolase b, fructose-bisphosphate (*aldob*), fructose-bisphosphate aldolase C (*aldoc*), phosphoglycerate mutase 1 (*pgam1*), alpha-enolase (*eno1*), pyruvate kinase L/R (*pklr*), and lactate dehydrogenase Bb (*ldhbb*). Despite similar functions as *pfkm*, *aldoa*, *pgam2*, *eno3*, and *pkm*, which are mainly expressed in the muscle, *pfkp*, *aldob*, *aldoc*, *pgam1*, *eno1*, and *pklr* are mainly expressed in non-muscle tissues (e.g., brain, liver, blood) (Verma and Dutta, 1994; Zhang et al., 2001; Caspi et al., 2014; Ausina et al., 2018; Tarnopolsky, 2018; Ždralević et al., 2018). Their downregulation in the fast-growth group and upregulation in the slow-growth group may be a sign of energy homeostasis, whereby energy was supplied to the brain and liver to maintain normal vital activities in the slow-growth group, but excess energy was supplied to the muscles for growth in the fast-growth group.

Several down-regulated DEGs in the fast-growth group were also enriched in the peroxisome and PPAR signaling pathways, which mainly regulate fatty acid transport and *β* oxidation to degradation (Ordovás et al., 2006; Morais et al., 2007; Watkins et al., 2007; Fidaleo et al., 2011; Shinoda et al., 2020), including peroxisomal acyl-coenzyme A oxidase 1 (*acox1*), 3-ketoacyl-CoA thiolase B, peroxisomal (*acaa1*), very long-chain acyl-CoA synthetase (*slc27a2*), fatty acid binding protein 1-B.1 (*fabp1b.1*), and long-chain fatty acid transport protein 1 (*slc27a1*) (Figure 5). These results indicate that fatty acid utilization is lower in the faster growing fish. In addition, some DEGs involved in the regulation of cholesterol and polyunsaturated fatty acid synthesis were also down-regulated in the fast-growth group. Cholesterol and polyunsaturated fatty acids are extremely important biological molecules that play essential roles in membrane structure and are precursors for the synthesis of other biological molecules (Simons and Ikonen, 2000; Christie and Harwood, 2020). Endogenous cholesterol and polyunsaturated fatty acid biosynthesis are affected by existing intracellular levels, i.e., higher food intake leads to lower endogenous biosynthesis in the body, while lower food intake has the opposite effect (Simons and Ikonen, 2000; Xu et al., 2020). This suggests that the fast-growth group had a higher dietary intake of cholesterol and polyunsaturated fatty acids, and thus could not mobilize endogenous synthesis to meet body needs.

Amino acids can directly promote muscle growth in fish, both by stimulating rates of protein synthesis and reducing rates of protein degradation (Seiliez et al., 2008; Cleveland and Radler, 2019). In the current study, we identified several DEGs correlated with glycine, serine, arginine, and proline metabolism (Figure 5), e.g., serine-pyruvate aminotransferase, mitochondrial (*agxt*), serine hydroxymethyltransferase 1 (*shmt1*), glutamate-ammonia ligase (glutamine synthase) (*glula*), cystathionine gamma-lyase (*cth*), and prolyl 4-hydroxylase subunit alpha-1 (*p4ha1*). In the fast-growth group, *agxt*, *shmt1*, *cth*, and *glula* were down-regulated. *Agxt* encodes serine-pyruvate aminotransferase, which catalyzes the conversion of alanine and glyoxylate into pyruvate and glycine, respectively (Cellini et al., 2007; Montioli et al., 2015), *shmt1* catalyzes the transfer of serine to glycine (Wang et al., 2013; Pinthong et al., 2014), *cth* catalyzes the conversion of cystathionine into cysteine (Krück et al., 2009), and *glula* regulates *de novo* glutamine production from glutamate (Eelen et al., 2018). *P4ha1*, which catalyzes the formation of 4-hydroxyproline (Rappu et al., 2019; Tolonen et al., 2022), was up-regulated in the fast-growth group. These results indicate that the fast-growth group may exhibit stronger 4-hydroxyproline synthesis, lower glycine, cysteine, and glutamine synthesis, and somewhat lower amino acid synthesis ability than the slow-growth group.

Based on the expression of genes related to glucose, fatty acid, and amino acid metabolism, the fast-growth group exhibited higher glucose and lower fatty acid utilization and lower amino acid synthesis activity compared to the slow-growth group. These results suggest that the fast-growth group consumed adequate energy (glucose, fatty acid, and amino acid) from fodder, with excess energy substances used for growth after maintenance of normal life activities. Therefore, energy intake and metabolism are crucial for *S. grahami* growth. Energy intake may be the root cause for the gap in growth between fast- and slow-growing *S. grahami* fish. Thus, the genes that regulate appetite and food intake (e.g., *adipoq*) require further analysis.

The synthesis of macromolecular substances (e.g., protein) is the foundation for growth and is based on the uptake and metabolism of energy substances. Here, we identified several collagen synthesis genes with a high degree of node connection (degree ≥ 18) based on WGCNA, which allows exploration of the correlations among large-scale gene expression data and phenotypes (Langfelder and Horvath, 2008). These genes, including *col1a1*, *col1a2*, *col5a1*, *col6a2*, *col10a1*, and *col26a1*, were up-regulated in the fast-growth group and significantly positively correlated with body length/weight. Genes with a high degree of node connection are significantly correlated with proximity to the center of the network (Zhou et al., 2022). Thus, *col1a1*, *col1a2*, *col5a1*, *col6a2*, *col10a1*, and *col26a1* may be crucial genes for *S. grahami* growth. The upregulation of these genes implies that collagen synthesis ability is higher in faster growing fish (Tolonen et al., 2022). Collagen accounts for one-third of total protein in postnatal animals. It is the main component of connective tissue and plays an important role in force transmission and tissue structure maintenance, especially tendons, ligaments, bone, and muscle, as well as in growth, development, and health (KJÆR, 2004; Zhang et al., 2005; Li and Wu, 2018). Glycine, proline, and hydroxyproline are major amino acids, accounting for 57% of total amino acids in collagen (Li and Wu, 2018). In our study, *p4ha1* was up-regulated in the fast-growth group. This may promote collagen synthesis as *p4ha1* catalyzes 4-hydroxyproline formation, which is essential for proper three-dimensional folding of newly synthesized procollagen chains (Rappu et al., 2019; Tolonen et al., 2022). However, some genes related to glycine and proline synthesis were not markedly different or down-regulated in the fast-growth group. These results suggest that the fast-growth group consumes sufficient amino acids for direct utilization, thereby reducing the endogenous synthesis of amino acids, which is energy efficient and will promote body growth. The increased synthesis of 4-hydroxyproline but not glycine and proline may be because 4-hydroxyproline is produced from proline-containing collagen rather than from free amino acids (Gorres and Raines, 2010).

WGCNA also identified *bglap* and *krt15* as crucial genes with the largest expression differences in *S. grahami* growth (Figure 1E; Figure 3D). Studies have shown that *bglap* and *krt15* play critical roles in bone formation and mineralization and in structural integrity (Lee et al., 2007; Bose et al., 2013; Komori, 2020). We also identified several growth factors and muscle-growth related genes that were up-regulated in the fast-growth group, such as bone morphogenetic protein 2 (*bmp2*), bone morphogenetic protein 3 (*bmp3*), transforming growth factor beta-1 (*tgfb1*), transforming growth factor beta-2 (*tgfb2*), growth/differentiation factor 10 (*gdf10*), myogenin (*myog*), myosin-binding protein C, fast-type (*mybpc2*), myosin heavy chain, fast skeletal muscle (*myh*), myosin light chain, skeletal muscle (*myl1*, *myl3*), troponin I, fast skeletal muscle (*tnni2*), troponin T, fast skeletal muscle (*tnnt3*), and troponin C, skeletal muscle (*tnnc2*). Growth/differentiation factors (GDFs), BMPs, and TGF-β are multi-functional growth factors belonging to the TGF-β superfamily and play important roles in development and tissue homeostasis via regulation of cell proliferation, migration, and differentiation, ECM production, multiple cellular signal transduction, cardiogenesis, somite formation, neurogenesis, and musculoskeletal development (Cunningham et al., 1995; Macias et al., 1997; Hino et al., 2004; Maatouk et al., 2009; Heldin and Moustakas, 2016; Zhou et al., 2016). Studies have also found that TGF-β stimulates collagen synthesis and mediates metabolic pathways by regulating the expression of glucose transporter 1 (*slc2a1*) (Johnston and Gillis, 2017; Yu et al., 2019; Zhou et al., 2021). *Myog* belongs to the Myogenic Regulatory Factors (MRFs) family, plays a crucial role in myogenesis (De-Santis and Jerry, 2007). Myosin heavy chain and myosin light chain are major component for skeletal muscle myosins (Schiaffino and Reggiani, 1996). Troponin is the key calcium-dependent regulator of striated muscles, and composed of troponin C (TnC), troponin I (TnI), and troponin T (TnT) (Rasmussen and Jin, 2021). Myosin, troponin and myosin-binding protein C are crucial components of skeletal muscle, which essential for myogenesis, muscle contraction (Das et al., 2019; Song et al., 2021; Rasmussen and Jin, 2021). The high expression of these genes would strengthen the muscle growth and contraction.

The WGCNA results also indicated that the differences in collagen synthesis may be the direct cause of the growth gap between the fast- and slow-growth groups of *S. grahami*. Glycine, proline and hydroxyproline are the main amino acids for collagen synthesis. Endogenous amino acid synthesis (e.g., proline and hydroxyproline) consumes a large amount of adenosine triphosphate (ATP) but is inadequate to meet optimal growth and connective tissue repair (Li and Wu, 2018). Hence, adequate amounts of dietary proline and hydroxyproline are essential for maximizing growth performance and feed efficiency in farmed *S. grahami*.

## 5 Conclusion

Based on transcriptomic analysis of *S. grahami* muscle, we identified various genes related to glucose, fatty acid, and amino acid uptake and metabolism, and collagen synthesis, which play crucial roles in promoting bone and muscle growth. Energy uptake and collagen synthesis may be the key factors for the growth gap between fast- and slow-growth *S. grahami*, and energy uptake may be the root cause, while collagen synthesis may be the direct reason. The reasons for differences in uptake and how to improve intake and collagen synthesis require further research. Our findings provide new insights into the mechanism underlying the growth gap between fast- and slow-growth *S. grahami* and provide an important theoretical basis for guiding *S. grahami* breeding. Furthermore, these results may provide valuable information for further studying of growth mechanisms and breeding strategies in other species.

## Data Availability

The datasets presented in this study can be found in online repositories. The names of the repository/repositories and accession number(s) can be found below: https://www.ncbi.nlm.nih.gov/, PRJNA907410.
