# Supplementary material for: Muscle transcriptome analysis provides new insights into the growth gap between fast- and slow-growing Sinocyclocheilus grahami
Source: Front Genet. 2023 Jul 19;14:1217952. doi: 10.3389/fgene.2023.1217952 (PMC10394708; doi:10.3389/fgene.2023.1217952)
Supplement: Supplementary file 1 [file DataSheet1.docx]

**Supplementary Materials and Methods**

**Fish cultivation, sample collection and sequencing for preliminary study**

In February 2018, a sibling population (generated from one female × one male, with about 2,000 individuals) and a random population (generated from multiple females × males, with about 20,000 individuals) from the farmed “*S. grahami*, Bayou No. 1” were constructed independently using artificial reproduction techniques. The resulting offspring were cultivated in a 1.5 m × 1 m × 0.8 m tank and a 3 m × 4 m × 1.5 m pond, respectively. The rearing conditions were consistent between the two populations, with the water temperature, dissolved oxygen (DO) and PH levels maintained near 22 ± 1 °C, 6.5 mg/L and 8.0, respectively. One third of water in tank/pond was changed with fresh water daily. They were fed twice a day (fodder volume 3% of fish weight) to apparent satiation by a commercial diet (protein 40%, lipid 18%, Specialized and high-end feed for freshwater fish, Tongwei Group) for eight months at the EFCC. We then separated individuals of each population into large-, medium-, and small-sized groups (in different buckets) according to body size. And transported 500 individuals (200 individuals with extremely large size, 200 individuals with extremely small size, and 100 individuals with medium size) labeled with visible implant elastomer (VIE) of different colors from each population into two fish tanks (1m × 0.6m × 1m) in the laboratory of KIZ for a further cultivation to observe the growth. The rearing conditions and feeding regime were consistent with previous setup. After 2 months of regular feeding, we measured body weight and body length separately, and the growth gap between extremely large, medium-sized and extremely small individuals persists throughout the experiment. Subsequently, we selected 30 individuals with extremely large size and 30 individuals with extremely small size as two extreme bulks of growth in each population. Finally, within the two examined populations, four bulks were obtained.

Fish samples from the four bulks were firstly euthanized using MS-222. Muscle tissues of each individual were collected and kept under sterile conditions. Subsequently, Total RNA of each individual was extracted using an RNA Purification Kit (Omega BioTek, USA) in accordance with the manufacturer’s instructions. For each sample, RNA concentration and quality were measured using a Nanophotometer (Implen, Germany) and Agilent 2100 Bioanalyzer (Agilent Technologies Inc., USA), respectively. We collected the final bulk samples using a pooled strategy: 30 samples in the same bulk were mixed by extracted an equal amount of total RNA from each sample. In total, four pooled samples corresponding to the four bulks were obtained, and then these samples were labelled into a pair-end 150bp library and sequenced at an Illumina Hiseq X-Ten platform.

**Transcript-level gene expression analysis and functional enrichment of differentially expressed genes (DEGs) for preliminary study**

Raw RNA sequencing (RNA-seq) reads from each bulk sample were filtered using FastQC (v0.11.8) and Trimmomatic (v0.38). Clean reads were aligned to the reference genome (GenBank: GCA_001515645.1) using Hisat2 (v2.1.0) with default parameters. The unique mapped reads of each sample were used to calculate fragments per kilobase of exon model per million mapped fragments (FPKM) using Cufflinks (v2.2.1) with default settings. According to the gene expression level of each sample, package edgeR in R (v4.0.5) was used to detect significant DEGs, with |log2 (fold-change)| ≥ 1 and adjusted *p* ≤ 0.05 applied as filtering thresholds. To further clarify the functions of DEGs, Gene Ontology (GO) and Kyoto Encyclopedia of Genes and Genomes (KEGG) pathway enrichment analyses were performed using DAVID (https://david.ncifcrf.gov/summary.jsp) and the clusterProfiler package in R (v4.0.5).

**Simple results for preliminary study**

The body length and body weight for the extreme bulks in two populations were similar (Supplementary Figure S1). After quality trimming, a total of 1,369,052,738 (the clean reads for fast-growth bulk in sibling population, slow-growth bulk in sibling population, fast-growth bulk in random population and slow-growth bulk in random population is 333,339,588, 333,614,302, 350,861,850 and 351,236,998, respectively) clean reads (150 bp) were generated for analysis. In sibling population, 3,224 up-regulated genes and 5,703 down-regulated genes were identified in the fast-growth bulk. Similarly, 2,290 up-regulated genes and 4,592 down-regulated genes were identified in the fast-growth bulk of random population. Furthermore, we observed 839 genes that were commonly up-regulated and 2,521 genes that were commonly down-regulated in the fast-growth bulk of both sibling and random populations (Supplentary Figure S2 A and B). The KEGG pathway enrichment analysis showed that up-regulated genes of sibling population, random population and common between two populations were predominantly enriched in Focal adhesion, ECM-receptor interaction, Glycolysis/Gluconeogenesis, and Biosynthesis of amino acids. Conversely, down-regulated genes were primarily enriched in Cell adhesion molecules, PPAR signaling pathway, and Steroid biosynthesis (Supplementary Figure S2 C and D). These results indicate that the major differentially expressed genes (DEGs) were consistent between the two populations.

**Supplementary Tables**

**Table S1 Transcriptome data information for all samples**

| Sample | Raw Reads | Clean Reads | Raw Base(G) | Clean Base(G) | Effective Rate(%) | Error Rate(%) | Q20(%) | Q30(%) | GC Content(%) |
| --- | --- | --- | --- | --- | --- | --- | --- | --- | --- |
| Fast-1 | 28,778,594 | 27,910,911 | 8.63 | 8.37 | 96.98 | 0.02 | 98.56 | 95.59 | 49.42 |
| Fast-2 | 32,097,847 | 31,129,564 | 9.63 | 9.34 | 96.98 | 0.02 | 98.48 | 95.37 | 49.67 |
| Fast-3 | 28,508,173 | 27,470,587 | 8.55 | 8.24 | 96.36 | 0.02 | 98.56 | 95.59 | 50 |
| Fast-4 | 29,329,282 | 28,579,824 | 8.8 | 8.57 | 97.44 | 0.02 | 98.65 | 95.78 | 49.22 |
| Fast-5 | 28,509,178 | 27,623,257 | 8.55 | 8.29 | 96.89 | 0.02 | 98.56 | 95.56 | 49.8 |
| Fast-6 | 26,416,736 | 25,709,798 | 7.93 | 7.71 | 97.32 | 0.02 | 98.5 | 95.38 | 49.52 |
| Fast-7 | 28,918,590 | 28,035,319 | 8.68 | 8.41 | 96.95 | 0.02 | 98.62 | 95.75 | 49.03 |
| Fast-8 | 27,213,794 | 26,452,878 | 8.16 | 7.94 | 97.2 | 0.02 | 98.56 | 95.57 | 49.37 |
| Fast-9 | 27,207,563 | 26,294,654 | 8.16 | 7.89 | 96.64 | 0.02 | 98.62 | 95.76 | 49.61 |
| Fast-10 | 29,345,069 | 28,544,434 | 8.8 | 8.56 | 97.27 | 0.02 | 98.6 | 95.62 | 49.47 |
| Fast-11 | 27,918,240 | 27,062,117 | 8.38 | 8.12 | 96.93 | 0.02 | 98.52 | 95.42 | 49.81 |
| Fast-12 | 29,178,445 | 28,342,524 | 8.75 | 8.5 | 97.14 | 0.02 | 98.56 | 95.55 | 49.58 |
| Fast-13 | 28,927,190 | 28,144,440 | 8.68 | 8.44 | 97.29 | 0.02 | 98.53 | 95.44 | 49.61 |
| Fast-14 | 29,739,523 | 28,756,945 | 8.92 | 8.63 | 96.7 | 0.02 | 98.62 | 95.68 | 49.72 |
| Slow-1 | 26,992,077 | 26,237,335 | 8.1 | 7.87 | 97.2 | 0.02 | 98.55 | 95.5 | 49.17 |
| Slow-2 | 28,416,384 | 27,524,920 | 8.52 | 8.26 | 96.86 | 0.02 | 98.61 | 95.65 | 48.52 |
| Slow-3 | 28,886,468 | 27,947,645 | 8.67 | 8.38 | 96.75 | 0.02 | 98.5 | 95.41 | 49.62 |
| Slow-4 | 29,498,533 | 28,591,680 | 8.85 | 8.58 | 96.93 | 0.02 | 98.61 | 95.67 | 49.11 |
| Slow-5 | 29,454,903 | 28,389,520 | 8.84 | 8.52 | 96.38 | 0.02 | 98.52 | 95.42 | 49.38 |
| Slow-6 | 28,693,496 | 27,746,134 | 8.61 | 8.32 | 96.7 | 0.02 | 98.53 | 95.45 | 48.33 |
| Slow-7 | 30,484,566 | 29,468,726 | 9.15 | 8.84 | 96.67 | 0.02 | 98.55 | 95.49 | 49.08 |
| Slow-8 | 27,660,724 | 26,824,971 | 8.3 | 8.05 | 96.98 | 0.02 | 98.44 | 95.25 | 49.2 |
| Slow-9 | 28,973,465 | 27,951,838 | 8.69 | 8.39 | 96.47 | 0.02 | 98.54 | 95.43 | 48.86 |
| Slow-10 | 27,028,987 | 26,232,999 | 8.11 | 7.87 | 97.06 | 0.02 | 98.54 | 95.5 | 49.14 |
| Slow-11 | 29,116,762 | 28,223,387 | 8.74 | 8.47 | 96.93 | 0.02 | 98.47 | 95.3 | 48.81 |
| Slow-12 | 30,177,749 | 29,130,454 | 9.05 | 8.74 | 96.53 | 0.02 | 98.51 | 95.46 | 49.24 |
| Slow-13 | 30,599,457 | 29,645,791 | 9.18 | 8.89 | 96.88 | 0.02 | 98.59 | 95.61 | 48.99 |
| Slow-14 | 28,077,233 | 27,208,418 | 8.42 | 8.16 | 96.91 | 0.02 | 98.5 | 95.42 | 48.84 |
| **Total** | **806,149,028** | **781,181,070** | **241.85** | **234.35** | **96.91** | **0.02** | **98.55** | **95.52** | **49.29** |

**Table S2 qRT-PCR primers’ information for 10 DEGs**

| Gene | 5'-3' primer sequence | | Product size (bp) |
| --- | --- | --- | --- |
| *col1a2* | Forward | AGTTGTCACTAGTTGGTACTAAGGT | 172 |
|  | Reverse | GGTTATCAGGGGGACCTGTG |  |
| *bglap* | Forward | TCCCAGTGATGATGGTGTGTTT | 145 |
|  | Reverse | CTCACAGGCCAGGTTTGCTT |  |
| *adipoq* | Forward | TGTGGATGGGAGGTGTTCCT | 118 |
|  | Reverse | TTCGCACCTGCTTCACCTTTAT |  |
| *col6a1* | Forward | CCAGGTTACCGTGGTGATGAA | 117 |
|  | Reverse | TGGTCCATCTTCACCCCTCTC |  |
| *col10a1* | Forward | TCCTACAAAATCTCACATGGTTGCT | 114 |
|  | Reverse | CCAGGGTGTCCAACTCCATTCTTT |  |
| *pgk1* | Forward | TTCGGGAACTTACCCAGCAC | 196 |
|  | Reverse | TGCAGCCTTGATTCTCTGGT |  |
| *pgm1* | Forward | AATTTATCAGGACCCACAGGTCA | 138 |
|  | Reverse | TGGATCAGGTGAATATGGCTCT |  |
| *aldoa* | Forward | TGCCATTAACCAGTGTCCCC | 160 |
|  | Reverse | GGCCAGGCTGTTGTTAAGAG |  |
| *krt15* | Forward | TCCAGACTCAAATACCAGGTGGA | 112 |
|  | Reverse | GCAGTGTGATGTGCATTTAGCAT |  |
| *cth* | Forward | AATCAAGAGCAATGGCACCAC | 197 |
|  | Reverse | CCGGTTTAGAACCCAACTACT |  |

**Supplementary Figures**

**
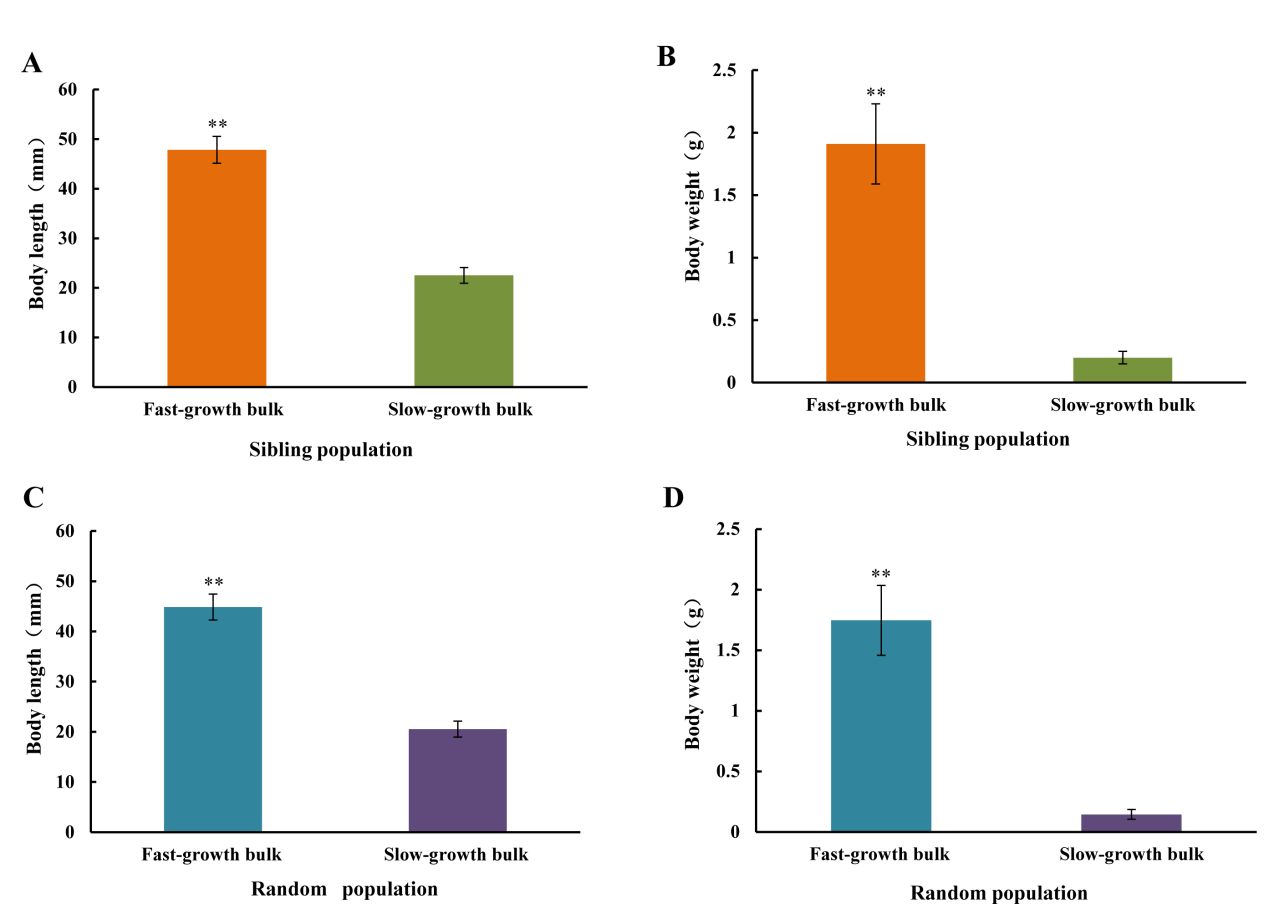
**

**Figure S1** **Phenotypic variation in the extreme bulk**. Body length (A) and body weight (B) variations in the sibling population; Body length (C) and body weight (D) variations in the random population. All the variations were statistically significant (**, *p* < 0.01).


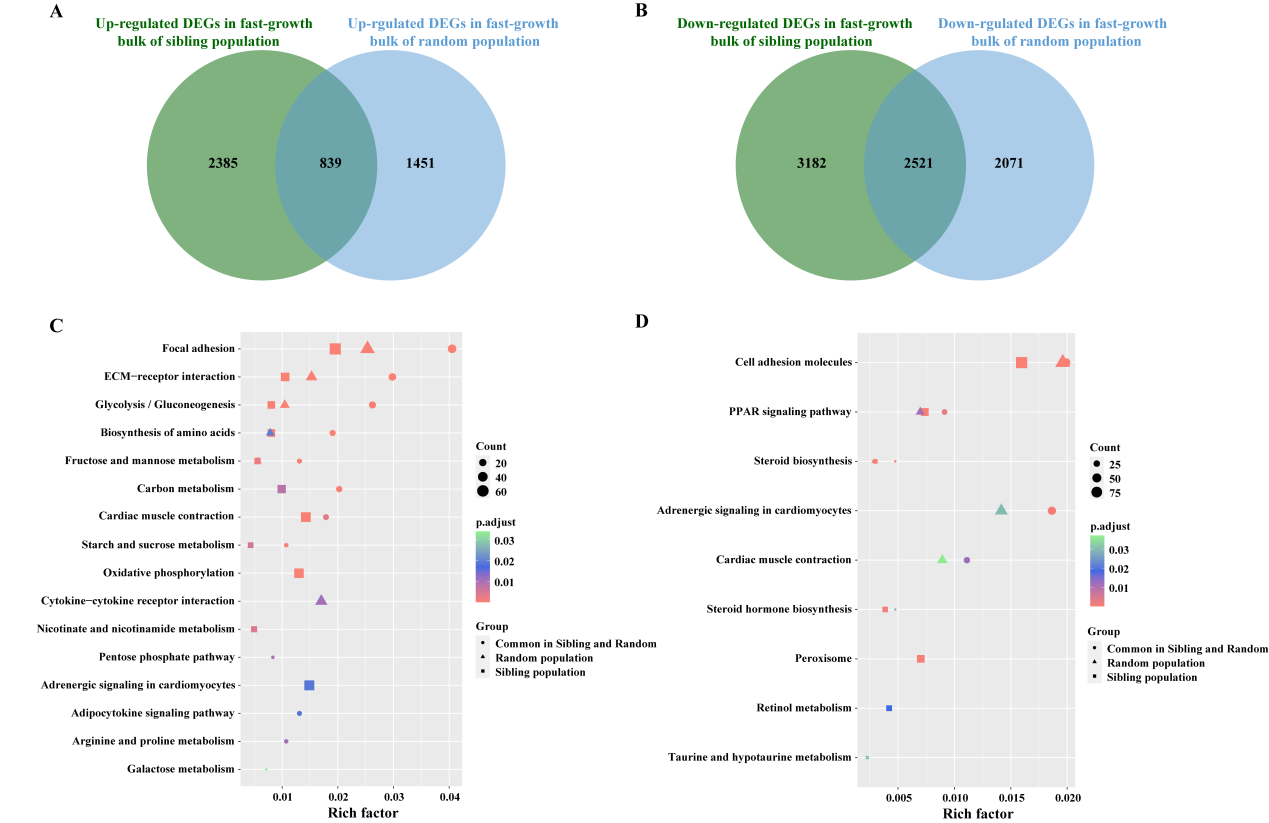


**Figure S2 DEGs number and KEGG enrichment for sibling and random population.** A. Venn diagram for up-regulated DEGs in fast-growth bulk of sibling and random population. B. Venn diagram for down-regulated DEGs in fast-growth bulk of sibling and random population. C. KEGG enrichment for up-regulated DEGs in fast-growth bulk of sibling population, random population and common in sibling and random population. D. KEGG enrichment for down-regulated DEGs in fast-growth bulk of sibling population, random population and common in sibling and random population.


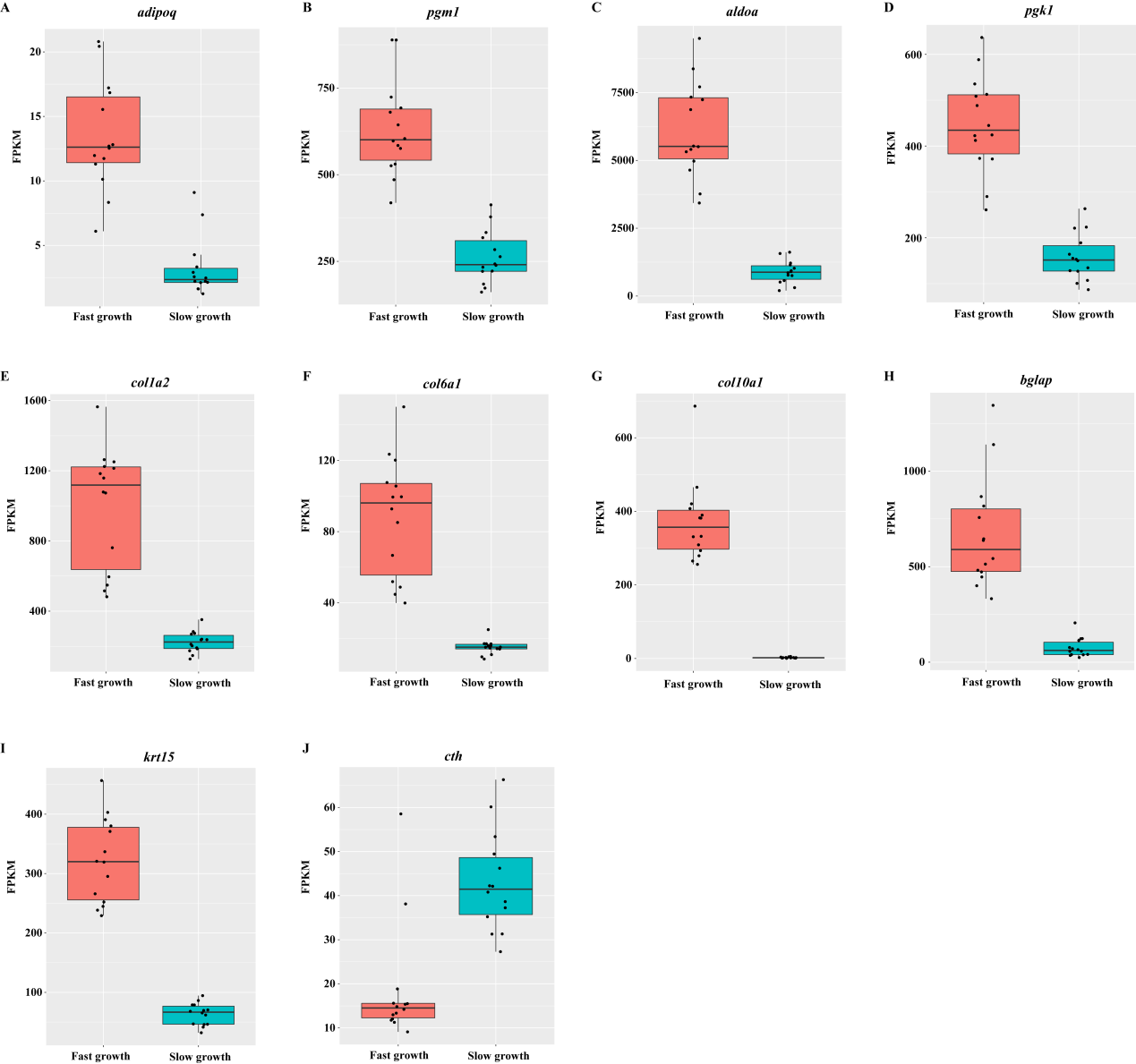


**Figure S3 The FPKM for 10 DEGs generated by RNA-seq.**

The box plot for the FPKM of *adipoq*, *pgm1*, *aldoa*, *pgk1*, *col1a2*, *col6a1*, *col10a1*, *bglap*, *krt15*, *cth* generated by RNA-seq are depicted in A, B, C, D, E, F, G, H, I, respectively. The red and cyan boxes represent the fast- and slow-growth groups, respectively. Each black dot represents the FPKM of a sample.


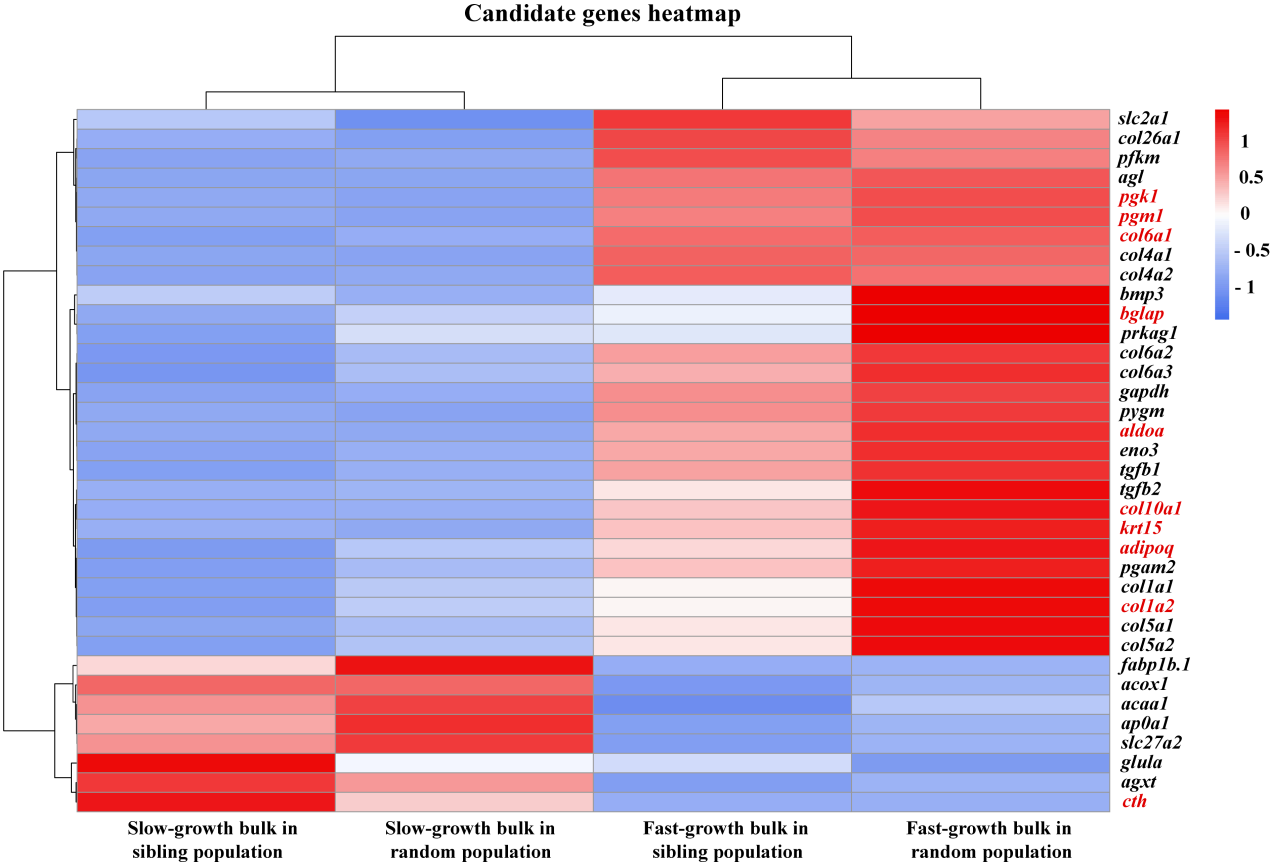


**Figure S4** **Heatmap for FPKM of candidate genes in bulk sample of sibling and random populations for preliminary study.** The genes correspond to the qRT-PCR were labeled with red color.
